# Uncovering the inflammatory profile of obese children: examining the link between body mass index for age and insulin resistance in the Gaza Strip

**DOI:** 10.3389/fped.2025.1570803

**Published:** 2025-06-26

**Authors:** Balsam Said Abu Ghazza, Abdel Hamid El Bilbeisi, Amany El Afifi

**Affiliations:** ^1^ Master Program of Clinical Nutrition, Faculty of Pharmacy, Al Azhar University of Gaza, Gaza Strip, Palestine; ^2^ Department of Clinical Nutrition, Faculty of Pharmacy, Al Azhar University of Gaza, Gaza Strip, Palestine; ^3^ Department of Nutrition, School of Medicine and Health Sciences, University of Palestine, Gaza Strip, Palestine; ^4^ Faculty of Pharmacy, Al Azhar University of Gaza, Gaza Strip, Palestine

**Keywords:** body mass index, children, Gaza Strip, inflammation, insulin resistance

## Abstract

**Objective:**

This study aimed to examine the role of inflammation in the relationship between body mass index (BMI)-for-age and insulin resistance among first-grade students in the Gaza Strip.

**Materials and methods:**

A cross-sectional study conducted between March and April 2023 involved 185 students, aged six, from five primary schools. Data were collected via structured questionnaires covering demographics, socio-economic status, lifestyle, and medical history. Anthropometric measurements, including BMI-for-age percentile, waist and hip circumferences, and blood pressure, were recorded. Biochemical analyses measured inflammatory markers (Hs-CRP, IL-6, adiponectin), fasting insulin, fasting plasma glucose, HDL-c, triglycerides, hemoglobin, and HOMA-IR. Statistical analysis was performed using SPSS version 25.

**Results:**

The study found that 74.6% of students had normal BMI-for-age, while 14.1% were overweight, 8.1% obese, and 3.2% underweight. BMI categories were significantly associated with socio-economic factors, especially place of residence and paternal education. Paternal employment was linked to insulin resistance. A significant association was found between BMI-for-age and insulin resistance, with higher insulin resistance in overweight and obese children. Waist and hip circumferences were significantly linked to BMI and insulin resistance. Hs-CRP and fasting insulin were significantly associated with insulin resistance (*P* values > 0.05 for all).

**Conclusion:**

The study confirms that childhood obesity is closely associated with both inflammation and insulin resistance. Elevated levels of IL-6, Hs-CRP, and insulin in obese children highlight the metabolic risks of childhood obesity. Socio-economic factors, including paternal education and place of residence, significantly influence BMI-for-age, suggesting the need for socio-economic considerations in obesity prevention efforts.

## Introduction

Childhood obesity has emerged as a significant public health concern worldwide, with its prevalence rising in both developed and developing countries (1). The global prevalence of childhood obesity has been steadily rising, with nearly 340 million children and adolescents classified as overweight or obese (2). In the Gaza Strip, this trend has been driven by a combination of environmental, socioeconomic, and dietary factors (3).

Childhood obesity is associated with a range of health complications, including insulin resistance, which serves as a key precursor to type 2 diabetes and other metabolic disorders (4). One significant cluster of these obesity-related complications is pediatric metabolic syndrome, a condition characterized by a combination of abdominal obesity, hypertension, dyslipidemia, and impaired glucose metabolism. This syndrome not only reflects metabolic dysfunction in childhood but also serves as a predictor of future health issues, including type 2 diabetes and cardiovascular disease (5). Evidence suggests that children diagnosed with metabolic syndrome are more likely to carry these risk factors into adulthood, thereby significantly increasing their risk of developing cardiovascular conditions later in life. The early onset of these metabolic abnormalities underscores the importance of identifying and addressing contributing factors such as insulin resistance during childhood (6). Insulin resistance in children can lead to long-term health consequences, such as metabolic syndrome and cardiovascular diseases, highlighting the importance of understanding the contributing factors (5). Insulin resistance is a metabolic condition in which normal or elevated insulin levels fail to elicit the expected biological response, particularly in glucose metabolism (6). It is a significant risk factor for type 2 diabetes and is influenced by genetic and environmental factors, including poor diet, physical inactivity, and obesity (7). In children, obesity has emerged as the primary risk factor for insulin resistance, reflecting the complex relationship between excess adiposity and metabolic dysregulation (8). In this study, insulin resistance was assessed using the Homeostasis Model Assessment for Insulin Resistance (HOMA-IR), with a value greater than 3.16 used as the diagnostic threshold for identifying insulin resistance (9). Body Mass Index (BMI) is widely used to assess the nutritional status of children and adolescents. Specifically, BMI-for-age is a valuable tool for classifying children into weight status categories, such as underweight, normal weight, overweight, and obese. However, BMI does not fully reflect the metabolic disturbances associated with obesity (10). Consequently, examining biomarkers that indicate metabolic dysfunction is essential for better understanding the link between weight status and insulin resistance (11).

Inflammation is a key factor influencing insulin resistance. Obesity often leads to a chronic low-grade inflammatory state, marked by elevated levels of pro-inflammatory cytokines such as interleukin-6 (IL-6) and high-sensitivity C-reactive protein (Hs-CRP). These inflammatory markers interfere with insulin signaling, contributing to insulin resistance (12). While the connection between inflammation and obesity-related insulin resistance is well established, the specific mechanisms and the extent of inflammation's role in this relationship remain unclear (13).

In the context of the Gaza Strip, a region facing challenges like political instability, limited healthcare access, and food insecurity, childhood obesity and insulin resistance are pressing concerns (14). To the best of our knowledge, while the relationship between BMI and insulin resistance in children is gaining attention, there is limited research on the role of inflammation in this connection within the Gaza Strip population. Understanding how inflammation impact the link between BMI-for-age and insulin resistance is critical for developing targeted interventions to address obesity and its related metabolic disorders in this vulnerable group. Additionally, the findings may offer valuable insights into potential strategies for early intervention and the prevention of insulin resistance, particularly in regions where childhood obesity is on the rise and healthcare resources are limited. Therefore, the current study was conducted to investigate the role of inflammation in the association between BMI-for-age percentile and insulin resistance among first-grade students, aged six, in primary governmental schools in the Gaza Strip.

## Materials and methods

### Study design

This observational, descriptive, and analytical cross-sectional study aimed to investigate the role of inflammation in the association between BMI-for-age percentile and insulin resistance among first-grade students, aged six, in primary governmental schools in the Gaza Strip.

### Study setting and period

The study took place between March 25 and April 27, 2023, before the Gaza war, and involved first-grade students from primary schools in the five Gaza governorates.

### Study population

The study focused on six-year-old first-grade students of both genders from primary schools across the five Gaza governorates: North Gaza, Gaza City, Middle Area, Khan Yunis, and Rafah Governorates.

### Eligibility criteria

In the current study, the inclusion criteria consisted of six-year-old first-grade students of both genders who were healthy, free from mental disabilities, and had parental consent. In contrast, children with psychiatric disorders, chronic illnesses, diabetes, medications affecting weight, endocrine or inflammatory conditions, and twins were excluded.

### Sample size and sampling technique

**Sample size:** According to a recent report from the Palestinian Ministry of Education, the projected number of first-grade students in governmental primary schools across the five Gaza governorates was approximately 22,917 (15). In this study, a representative sample of 185 first-grade students from primary governmental schools was selected, with the sample size calculated using the Charan and Biswas formula (16).

**Sample technique:** After applying the inclusion and exclusion criteria, a total of 185 six-year-old participants of both genders were proportionally selected from first-grade students at the five main primary governmental schools across the Gaza Strip's five governorates, based on population density. The distribution was as follows: 31 from North Gaza, 75 from Gaza City, 36 from the Middle Area, 28 from Khan Yunis, and 15 from Rafah.

### Data collection

#### Interview-based questionnaire

A pre-tested, interview-based questionnaire collected data on demographic, socio-economic, medical history, lifestyle factors, anthropometric, and biochemical measurements from the study participants.

#### Demographic socio-economic variables

The demographic and socio-economic section of the questionnaire collected information on family background, including the mother's and child's details, school stage, family income, parental employment, residence, education levels, and family size. This data was crucial for interpreting the study's results.

#### Medical history variables

The medical history section of the questionnaire collected data on the child's health, including any medical conditions, food allergies, weight changes, and appetite. It also reviewed maternal health during pregnancy, asking about complications like hypertension or gestational diabetes. This information provided key insights into the child's health.

#### Life style variables (breastfeeding, physical activity and nutritional behavior)

The lifestyle section of the questionnaire gathered data on infant feeding practices, including breastfeeding duration, and identified the family member responsible for meals. It also asked about the child's daily meals and snacks, providing insights into nutritional behavior and family involvement in feeding.

#### Assessment of physical activity

The lifestyle section of the questionnaire assessed physical activity using the physical activity questionnaire for children (PAQ-C), which measures activity over the past week. It covers various activities, including sports and physical education. The responses are scored on a 5-point scale, with the final score indicating the child's physical activity level. Based on this, children were classified into low, moderate, or high physical activity levels (17).

#### Assessment of dietary intakes and nutritional behavior

The Food Frequency Questionnaire (FFQ), validated in Arabic and adapted for the Palestinian context, was used to assess the children's food consumption over the past year (18, 19). Parents provided information on food groups like carbohydrates, vegetables, fruits, meats, dairy, beverages, and snacks. Responses were rated on a 4-point Likert scale from 1 (“Once a day”) to 4 (“Do not consume”). Based on the total score for 13 items, children were categorized as having Unhealthy (score < 60%), Moderate (score 60%–79%), or Healthy (score 80%–100%) eating practices, following Bloom's Taxonomy (20, 21).

#### Anthropometric measurements

**Weight (kg):** Children's weight was measured using a digital scale (SECA, Germany), with two readings taken and averaged for accuracy. The scale was calibrated at the start and end of each day, with adjustments made if calibration errors exceeded 0.1 kg. Weight was interpreted using age- and sex-specific World Health Organization (WHO) growth reference percentiles for children aged 5 to 19 years (22).

**Height (cm):** Children's height was measured using a stadiometer, following standard procedures, to the nearest 0.5 cm. Two measurements were taken, and the average was used. The height data were also evaluated according to WHO age- and sex-specific growth percentiles (23).

**Body Mass Index (kg/m^2^):** BMI was calculated by dividing weight in kilograms by height in meters squared. The BMI-for-age percentiles were determined using the WHO AnthroPlus software, which classifies children into underweight (<5th percentile), normal weight (5th–84.9th percentile), overweight (85th–94.9th percentile), and obese (≥95th percentile) (24).

**Waist circumference (cm):** Waist circumference was measured with a stretch-resistant tape, with children standing relaxed and breathing normally. The tape was placed at the midpoint between the rib and iliac crest, and the measurement was recorded after the child exhaled. The measurement was taken twice, and the average was used (25).

**Hip circumference (cm):** Hip circumference was measured with a stretch-resistant tape at the level of the greater trochanter. The child stood relaxed with feet apart, and the measurement was taken after ensuring the tape was level and the child was breathing normally (26). The measurement was recorded twice, with the average as the final result.

**Blood pressure (mmHg):** Blood pressure was measured three times using a mercury sphygmomanometer in a quiet setting, with the child seated and relaxed. The average of the three readings was recorded. Blood pressure values were interpreted using age-, sex-, and height-specific percentiles based on international pediatric blood pressure reference charts, with elevated blood pressure defined as systolic or diastolic blood pressure ≥ the 90th percentile for age, sex, and height (27).

#### Child's biochemical measurements

Venous blood samples were collected after 12 h of fasting for various biochemical analyses, including: Fasting insulin (µU/ml), fasting plasma glucose (mg/dl), hemoglobin (g/dl), interlukin_6 (pg/ml), adiponectin (µg/ml), high-sensitivity C-reactive protein (Hs CRP) (mg/L), high-density lipoprotein-cholesterol (HDL-c) (mg/dl), and triglyceride (mg/dl). The tests were performed using a Mindray BS-300 chemistry analyzer with ELISA kits, and the processing was done at a licensed private laboratory.

**Fasting plasma glucose (mg/dl):** In healthy six-year-old children, the typical range for normal fasting blood glucose is below 100 mg/dl. A level between 100 and 125 mg/dl is considered impaired fasting glucose, often referred to as prediabetes. Levels above 126 mg/dl usually indicate diabetes (28).

**Hemoglobin (g/dl):** In healthy six-year-old children, the normal hemoglobin level typically ranges between 11.5 and 15.5 g/dl (29).

**Interlukin_6 (pg/ml):** In healthy six-year-old children, IL-6 levels are typically below 10 pg/ml. Levels above 10 pg/ml can indicate inflammation, as seen in conditions like obesity, chronic inflammation, or sepsis (30).

**Adiponectin (µg/ml):** In healthy six-year-old children, adiponectin levels typically range from 3 to 30 µg/ml. Lower levels are often seen in children with higher BMI and insulin resistance (31).

**Hs-CRP (mg/L):** In healthy six-year-old children, Hs-CRP levels are typically below 1 mg/L. Levels between 1 and 3 mg/L indicate minor infections or low-grade inflammation, often seen with viral infections or mild obesity-related inflammation (32).

**HDL-c (mg/dl):** In healthy six-year-old children, HDL-c levels are typically above 45 mg/dl, as it is considered the “good” cholesterol (33).

**Triglyceride (mg/dl):** In healthy six-year-old children, normal triglyceride levels are below 75 mg/dl. Levels between 75 and 99 mg/dl are borderline high, and levels above 100 mg/dl are considered high (34).

**Homeostasis Model Assessment of Insulin Resistance (HOMA-IR):** In the current study, HOMA-IR was calculated to define insulin resistance using the following formula: HOMA-IR = (Fasting plasma glucose × Fasting insulin)/405 (35). For healthy six-year-old children, a HOMA-IR >3.16 is considered the diagnostic threshold for insulin resistance (9).

#### Pilot study

A pilot study involving twenty participants was conducted to evaluate the questionnaire and data collection methods. Feedback from this pilot led to adjustments in the questionnaire to improve clarity and accuracy for the main study.

#### Statistical analysis

Statistical analysis was performed using the Statistical Package for Social Sciences (SPSS) version 25. Data are expressed as means ± standard deviations (SD) for continuous variables and as percentages for categorical variables. Differences between means were tested using the independent sample *t*-test and one-way ANOVA. The chi-square test was used to examine differences in the prevalence of categorical variables. A *P*-value of less than 0.05 was considered statistically significant.

## Results

The study, involving 185 six-year-old children, with 43.8% males and 56.2% females. Most families (98.4%) had a monthly income below 1974 NIS, and a higher percentage of fathers (17.8%) were employed compared to mothers (2.2%). Most children (84.3%) lived in urban areas, particularly Gaza City (40.5%). In terms of BMI-for age, 74.6% were of normal weight, 14.1% overweight, 8.1% obese, and 3.2% underweight. No significant relationship was found between gender, family income, parental employment, or the number of family members and BMI-for age categories. However, significant differences were noted in BMI based on place of residence (*P* = 0.001), governorate (*P* = 0.008), and the father's education level (*P* = 0.047). Obesity was more common in cities, especially Gaza City, while underweight was more prevalent in villages, particularly Khan Younis. The father's education level was associated with BMI, with children of illiterate fathers having higher obesity rates. The mother's education level did not show a significant association with BMI. These findings highlight the influence of socio-economic factors, particularly place of residence and paternal education, on childhood BMI-for age as shown in Table 1.

**Table 1:** The relationship between demographic socio-economic variables and body mass index for age categories of the study participants.

Variables	Underweight *n* (%) 6.0 (3.2)	Normal weight *n* (%) 138 (74.6)	Overweight *n* (%) 26 (14.1)	Obese *n* (%) 15 (8.1)	*P* Value
Child's age (years)					
Six years	6.0 (3.2)	138 (74.6)	26 (14.1)	15 (8.1)	–
The child's school stage					
The first stage	6.0 (3.2)	138 (74.6)	26 (14.1)	15 (8.1)	–
Gender					
Male	2.0 (33.3)	56 (40.6)	14 (53.8)	9.0 (60.0)	0.319
Female	4.0 (66.7)	82 (59.4)	12 (46.2)	6.0 (40.0)	
Family monthly income in New Israeli Shekels (NIS)					
Less than 1,974 NIS	6.0 (3.3)	136 (74.7)	25 (13.7)	15 (8.3)	0.758
1,975–2,470 NIS	0.0 (0.0)	2.0 (66.7)	1.0 (33.3)	0.0 (0.0)	
Father's job					
Employee	1.0 (3.0)	24 (72.7)	3.0 (9.1)	5.0 (15.2)	0.364
Doesn’t work	5.0 (3.3)	114 (75.0)	23 (15.1)	10 (6.6)	
Mother's job					
Employee	0.0 (0.0)	3.0 (75.0)	0.0 (0.0)	1.0 (25.0)	0.543
Doesn’t work	6.0 (3.3)	135 (74.6)	26 (14.4)	14 (7.7)	
Place of residence					
City	1.0 (0.6)	119 (76.3)	21 (13.5)	15 (9.6)	**0**.**001**
Village	5.0 (17.2)	19 (65.6)	5.0 (17.2)	0.0 (0.0)	
Governorate					
North Gaza	0.0 (0.0)	24 (77.4)	5.0 (16.1)	2.0 (6.5)	**0**.**008**
Gaza City	0.0 (0.0)	58 (77.3)	9.0 (12.0)	8.0 (10.7)	
Middle Area	1.0 (2.8)	26 (72.2)	5.0 (13.9)	4.0 (11.1)	
Khan Younis	5.0 (17.9)	18 (64.2)	5.0 (17.9)	0.0 (0.0)	
Rafah	0.0 (0.0)	12 (80.0)	2.0 (13.3)	1.0 (6.7)	
Number of family member's					
Mean ± SD	5.6 ± 1.3	6.2 ± 1.7	6.3 ± 1.7	6.0 ± 1.7	0.805
Number of children less than five year's					
Mean ± SD	0.8 ± 0.4	1.0 ± 0.8	1.3 ± 0.7	1.2 ± 1.2	0.417
Father's educational level					
Illiterate	0.0 (0.0)	1.0 (33.3)	0.0 (0.0)	2.0 (66.7)	**0**.**047**
Primary	1.0 (5.3)	14 (73.7)	1.0 (5.3)	3.0 (15.7)	
Preparatory	0.0 (0.0)	27 (81.8)	5.0 (15.2)	1.0 (3.0)	
Secondary	4.0 (5.2)	57 (74.0)	12 (15.6)	4.0 (5.2)	
Diploma or university	1.0 (1.9)	39 (73.6)	8.0 (15.1)	5.0 (9.4)	
Mother's educational level					
Illiterate	0.0 (0.0)	1.0 (100)	0.0 (0.0)	0.0 (0.0)	0.325
Primary	0.0 (0.0)	1.0 (50.0)	1.0 (50.0)	0.0 (0.0)	
Preparatory	0.0 (0.0)	16 (69.6)	6.0 (26.1)	1.0 (4.3)	
Secondary	3.0 (3.1)	77 (81.1)	6.0 (6.3)	9.0 (9.5)	
Diploma or university	3.0 (4.7)	43 (67.2)	13 (20.3)	5.0 (7.8)	

Data are presented as means ± SD for continuous variables and percentages for categorical variables. Differences between means were assessed using One-Way ANOVA, and the Chi-square test was used to examine differences in the prevalence of categorical variables. The bold values indicated that the *P*-value less than 0.05 was considered statistically significant. SD stands for standard deviation. Underweight is defined as BMI-for-age below the 5th percentile, normal weight as BMI-for-age between the 5th and 85th percentiles, overweight as BMI-for-age between the 85th and 95th percentiles, and obesity as BMI-for-age above the 95th percentile.

Table 2 reveals significant differences across BMI categories in weight, height, waist circumference, and hip circumference, with weight showing a progressive increase from underweight (18.3 ± 1.1 kg) to obese children (29.6 ± 6.1 kg) and significant differences in height (*P* = 0.002), waist (*P* = 0.001), and hip circumference (*P* = 0.001). However, no significant differences were found in systolic (*P* = 0.427) or diastolic (*P* = 0.300) blood pressure across BMI categories. Additionally, no significant associations were found between BMI-for-age and variables such as pathological problems, food allergies, recent weight changes, or type of delivery (*P* > 0.05). Children with poor appetite showed a slightly higher obesity prevalence (13.8%), but this was not statistically significant (*P* = 0.275). Pregnancy complications, childbirth issues, and birth weight did not significantly affect BMI-for-age, although mean birth weight increased from underweight to obese categories without statistical significance (*P* = 0.307). These findings suggest that medical history factors did not significantly influence BMI-for-age in this sample.

**Table 2:** The relationship between anthropometric measurements and medical history variables and body mass index for age categories of the study participants.

Variables	Underweight *n* (%) 6.0 (3.2)	Normal weight *n* (%) 138 (74.6)	Overweight *n* (%) 26 (14.1)	Obese *n* (%) 15 (8.1)	*P* Value
Weight (kg)					
Mean ± SD	18.3 ± 1.1	20.7 ± 1.8	23.3 ± 1.8	29.6 ± 6.1	**0** **.** **001**
Height (cm)					
Mean ± SD	118.7 ± 3.9	115.4 ± 5.5	114.7 ± 3.9	120.6 ± 3.6	**0** **.** **002**
Waist circumference (cm)					
Mean ± SD	48.4 ± 1.4	51.2 ± 3.0	53.1 ± 3.4	60.6 ± 6.2	**0** **.** **001**
Hip circumference (cm)					
Mean ± SD	55.3 ± 2.7	60.5 ± 3.4	63.2 ± 2.7	70.4 ± 7.4	**0** **.** **001**
Systolic blood pressure (mmHg)					
Mean ± SD	101.5 ± 12.2	106.1 ± 15.2	111.0 ± 28.9	110.2 ± 14.6	0.427
Diastolic blood pressure (mmHg)					
Mean ± SD	59.0 ± 10.5	60.0 ± 10.5	61.1 ± 14.7	65.8 ± 11.9	0.300
Does your child suffer from any pathological problems?					
No	6.0 (3.2)	138 (74.6)	26 (14.1)	15 (8.1)	–
Does your child have a food allergy?					
No	6.0 (3.2)	138 (74.6)	26 (14.1)	15 (8.1)	–
Has your child suffered from noticeable gain or loss in weight lately?					
No	6.0 (3.2)	138 (74.6)	26 (14.1)	15 (8.1)	–
How would you describe a child's appetite for food?					
Good	0.0 (0.0)	4.0 (100)	0.0 (0.0)	0.0 (0.0)	0.275
Acceptable	6.0 (4.9)	92 (74.8)	18 (14.6)	7.0 (5.7)	
Poor	0.0 (0.0)	42 (72.4)	8.0 (13.8)	8.0 (13.8)	
During pregnancy in (child's name), did you suffer from dangerous pregnancy?					
Hypertension	0.0 (0.0)	5.0 (71.4)	2.0 (28.6)	0.0 (0.0)	0.817
Gestational diabetes	0.0 (0.0)	3.0 (75.0)	0.0 (0.0)	1.0 (25.0)	
Eclampsia	0.0 (0.0)	3.0 (75.0)	0.0 (0.0)	1.0 (25.0)	
Preterm delivery	0.0 (0.0)	3.0 (60.0)	1.0 (20.0)	1.0 (20.0)	
No suffering	6.0 (3.6)	124 (75.2)	23 (13.9)	12 (7.3)	
Type of the child's delivery					
Caesarean	1.0 (2.7)	26 (70.3)	6.0 (16.2)	4.0 (10.8)	0.867
Normal	5.0 (3.4)	112 (75.7)	20 (13.5)	11 (7.4)	
Have you experienced complications from childbirth, such as weak labor, uterine rupture, stitches, or others (in the delivery of the child)?					
Yes	5.0 (6.8)	53 (71.5)	11 (14.9)	5.0 (6.8)	0.159
No	1.0 (0.9)	85 (76.6)	15 (13.5)	10 (9.0)	
Childs birth weight (kg)					
Mean ± SD	2.8 ± 0.7	3.1 ± 0.4	3.0 ± 0.7	3.2 ± 0.6	0.307
Childs birth weight (categories)					
Low birth weight	1.0 (6.7)	9.0 (60.0)	4.0 (26.6)	1.0 (6.7)	0.255
Normal birth weight	5.0 (3.0)	128 (76.2)	22 (13.1)	13 (7.7)	
Large baby	0.0 (0.0)	1.0 (50.0)	0.0 (0.0)	1.0 (50.0)	
After birth was the child put the nursery for any reason?					
Yes	1.0 (5.6)	12 (66.6)	4.0 (22.2)	1.0 (5.6)	0.659
No	5.0 (3.0)	126 (75.4)	22 (13.2)	14 (8.4)	

Data are presented as means ± SD for continuous variables and percentages for categorical variables. Differences between means were tested using One-Way ANOVA, with a *P*-value less than 0.05 considered statistically significant (in bold). SD stands for standard deviation. BMI-for-age categories are defined as: underweight (<5th percentile), normal weight (5th–85th percentiles), overweight (85th–95th percentiles), and obese (>95th percentile). Definitions for other variables include preterm delivery (<37 weeks), low birth weight (<2,500 g), normal birth weight (2,500–4,000 g), and large baby (>4,000 g).

Table 3 found no statistically significant associations between various life style variables and BMI categories, as all *P*-values exceeded 0.05. Most participants were breastfed naturally, with a higher percentage in the normal weight category. Formula-fed children had a slightly higher prevalence of overweight, but this was not statistically significant. Exclusive breastfeeding for over six months was linked to a lower overweight rate compared to mixed feeding. The duration of breastfeeding had no significant effect on BMI categories, although longer breastfeeding durations were associated with higher mean BMI values. Feeding practices and meal frequency showed no significant relationship with BMI. Physical activity levels did not show a significant correlation with BMI; however, children with higher levels of physical activity had a higher prevalence of overweight, whereas those with lower activity levels had a lower prevalence (*P* = 0.263).

**Table 3:** The relationship between life style variables (breastfeeding, and physical activity levels) and body mass index for age categories of the study participants.

Variables	Underweight *n* (%) 6.0 (3.2)	Normal weight *n* (%) 138 (74.6)	Overweight *n* (%) 26 (14.1)	Obese *n* (%) 15 (8.1)	*P* Value
Did you breastfeed your child naturally?					
Yes	5.0 (2.8)	135 (75.4)	24 (13.4)	15 (8.4)	0.107
No	1.0 (16.7)	3.0 (50.0)	2.0 (33.3)	0.0 (0.0)	
Was breastfeeding only?					
Yes	2.0 (1.7)	91 (77.1)	13 (11.0)	12 (10.2)	0.088
No	4.0 (6.0)	47 (70.1)	13 (19.4)	3.0 (4.5)	
Duration of breastfeeding (months)					
Mean ± SD	10.6 ± 7.3	13.5 ± 5.2	12.8 ± 6.0	14.4 ± 5.8	0.497
Duration of breastfeeding (categories)					
Less than six months	2.0 (13.3)	9.0 (60.0)	3.0 (20.0)	1.0 (6.7)	0.390
Six to twelve months	1.0 (3.7)	20 (74.1)	4.0 (14.8)	2.0 (7.4)	
More than 12 months	3.0 (2.1)	109 (76.2)	19 (13.3)	12 (8.4)	
Type of child feeding					
Formula fed only	1.0 (16.7)	3.0 (50.0)	2.0 (33.3)	0.0 (0.0)	0.113
Breastfeeding & formula fed	3.0 (4.9)	44 (72.2)	11 (18.0)	3.0 (4.9)	
Exclusive breastfeeding	2.0 (1.7)	91 (77.1)	13 (11.0)	12 (10.2)	
Who is responsible for preparing the food for the child?					
Mother	5.0 (5.2)	68 (70.1)	14 (14.4)	10 (10.3)	0.429
Father	0.0 (0.0)	27 (79.4)	7.0 (20.6)	0.0 (0.0)	
Sister	1.0 (2.6)	30 (78.9)	3.0 (7.9)	4.0 (10.6)	
Brother	0.0 (0.0)	13 (81.3)	2.0 (12.5)	1.0 (6.2)	
How many meals does your child eat per day?					
Mean ± SD	2.8 ± 0.4	2.8 ± 0.6	2.8 ± 0.7	2.8 ± 0.8	0.989
Regular meals categories					
Two regular meals or less	1.0 (1.9)	39 (73.6)	8.0 (15.1)	5.0 (9.4)	0.876
Three regular meals	5.0 (4.3)	88 (75.9)	15 (12.9)	8.0 (6.9)	
Four regular meal or more	0.0 (0.0)	11 (68.8)	3.0 (18.8)	2.0 (12.4)	
How many in-between meals does your child eat per day?					
Mean ± SD	2.1 ± 0.7	1.8 ± 1.1	1.8 ± 1.6	1.8 ± 0.9	0.904
Physical activity level categories					
Low physical activity level	1.0 (1.4)	58 (79.5)	8.0 (11.0)	6.0 (8.1)	0.263
Moderate physical activity level	4.0 (4.5)	66 (74.1)	11 (12.4)	8.0 (9.0)	
High physical activity level	1.0 (4.3)	14 (60.9)	7.0 (30.5)	1.0 (4.3)	

Data are expressed as means ± SD for continuous variables and as percentage for categorical variables. The differences between means were tested by using One-Way ANOVA. The Chi-square test was used to examine differences in the prevalence of different categorical variable. *P*-value less than 0.05 was considered as statistically significant. SD, stander deviation; Exclusive breastfeeding, more than six months.

Table 4 examines eating habits across various food groups and finds no statistically significant associations between individual food groups and BMI-for-age categories (*P*-values ranging from 0.118 to 0.886). However, trends showed that children in the underweight category had distinct eating patterns, such as higher adherence to healthy eating practices for vegetables and salads (5.3%). Despite this, no significant differences were found in fast food or fruit consumption. When assessing overall eating practices, a significant difference in the average eating practice score was found (*P* = 0.025), with the underweight category reporting the highest score (2.3 ± 0.2) compared to other BMI categories. Unhealthy eating practices were most common in the normal weight group. These findings suggest that while individual food group consumption did not correlate with BMI-for-age, the underweight group reported better overall eating habits.

**Table 4:** The relationship between eating practices using the food frequency questionnaire and body mass index for age categories of the study participants.

Items/food groups per week	Underweight *n* (%) 6.0 (3.2)	Normal weight *n* (%) 138 (74.6)	Overweight *n* (%) 26 (14.1)	Obese *n* (%) 15 (8.1)	*P* Value
Vegetables\salads					
Unhealthy eating practice	0.0 (0.0)	21 (87.5)	2.0 (8.3)	1.0 (4.2)	0.375
Moderate healthy eating practice	0.0 (0.0)	37 (77.1)	8.0 (16.7)	3.0 (6.2)	
Healthy eating practice	6.0 (5.3)	80 (70.8)	16 (14.2)	11 (9.7)	
Fruits					
Unhealthy eating practice	1.0 (5.6)	9.0 (50.0)	6.0 (33.3)	2.0 (11.1)	0.118
Moderate healthy eating practice	3.0 (4.4)	51 (73.9)	11 (15.9)	4.0 (5.8)	
Healthy eating practice	2.0 (2.0)	78 (79.6)	9.0 (9.2)	9.0 (9.2)	
Fast food					
Unhealthy eating practice	1.0 (1.3)	58 (80.6)	9.0 (12.5)	4.0 (5.6)	0.182
Moderate healthy eating practice	2.0 (3.6)	47 (79.7)	7.0 (11.9)	3.0 (5.1)	
Healthy eating practice	3.0 (5.6)	33 (61.1)	10 (18.5)	8.0 (14.8)	
Milk, cheese and dairy products					
Unhealthy eating practice	1.0 (1.4)	57 (77.0)	10 (13.5)	6.0 (8.1)	0.686
Healthy eating practice	5.0 (4.5)	81 (73.0)	16 (14.4)	9.0 (8.1)	
Meat					
Unhealthy eating practice	1.0 (4.3)	20 (87.0)	2.0 (8.7)	0.0 (0.0)	0.557
Moderate healthy eating practice	2.0 (2.4)	64 (76.2)	10 (11.9)	8.0 (9.5)	
Healthy eating practice	3.0 (3.8)	54 (69.3)	14 (17.9)	7.0 (9.0)	
Fish					
Unhealthy eating practice	1.0 (1.8)	45 (78.9)	7.0 (12.3)	4.0 (7.0)	0.472
Moderate healthy eating practice	0.0 (0.0)	37 (74.0)	9.0 (18.0)	4.0 (8.0)	
Healthy eating practice	5.0 (6.4)	56 (71.8)	10 (12.8)	7.0 (9.0)	
Chicken					
Unhealthy eating practice	1.0 (7.7)	9.0 (69.2)	3.0 (23.1)	0.0 (0.0)	0.183
Moderate healthy eating practice	0.0 (0.0)	71 (79.8)	10 (11.2)	8.0 (9.0)	
Healthy eating practice	5.0 (6.0)	58 (69.9)	13 (15.7)	7.0 (8.4)	
Candies and sweets					
Unhealthy eating practice	2.0 (1.8)	85 (76.6)	15 (13.5)	9.0 (8.1)	0.673
Moderate healthy eating practice	3.0 (4.8)	46 (74.2)	9.0 (14.5)	4.0 (6.5)	
Healthy eating practice	1.0 (8.3)	7.0 (58.3)	2.0 (16.7)	2.0 (16.7)	
Nuts					
Unhealthy eating practice	0.0 (0.0)	36 (75.0)	7.0 (14.6)	5.0 (10.4)	0.792
Moderate healthy eating practice	3.0 (4.8)	45 (71.4)	10 (15.9)	5.0 (7.9)	
Healthy eating practice	3.0 (4.1)	57 (77.0)	9.0 (12.1)	5.0 (6.8)	
Chocolate					
Unhealthy eating practice	1.0 (2.0)	40 (80.0)	5.0 (10.0)	4.0 (8.0)	0.886
Moderate healthy eating practice	5.0 (4.2)	86 (71.7)	19 (15.8)	10 (8.3)	
Healthy eating practice	0.0 (0.0)	12 (80.0)	2.0 (13.3)	1.0 (6.7)	
Soft drinks					
Unhealthy eating practice	2.0 (1.6)	95 (75.4)	17 (13.5)	12 (9.5)	0.218
Healthy eating practice	4.0 (6.8)	43 (72.8)	9.0 (15.3)	3.0 (5.1)	
Packaged fruit juices					
Unhealthy eating practice	4.0 (2.9)	104 (76.5)	18 (13.2)	10 (7.4)	0.803
Healthy eating practice	2.0 (4.1)	34 (69.4)	8.0 (16.3)	5.0 (10.2)	
Legumes (chickpeas, beans, lentils)					
Unhealthy eating practice	2.0 (8.3)	17 (70.8)	3.0 (12.5)	2.0 (8.4)	0.634
Moderate healthy eating practice	2.0 (2.9)	56 (80.0)	8.0 (11.4)	4.0 (5.7)	
Healthy eating practice	2.0 (2.2)	65 (71.4)	15 (16.5)	9.0 (9.9)	
Mean eating practice					
Mean ± SD	2.3 ± 0.2	2.0 ± 0.2	2.0 ± 0.2	2.1 ± 0.1	**0** **.** **025**
Score eating practice					
Percentage (%)	0.76 ± 0.07	0.67 ± 0.08	0.69 ± 0.06	0.70 ± 0.06	**0** **.** **025**
Score eating practice categories					
Unhealthy eating practice	0.0 (0.0)	25 (83.3)	3.0 (10.0)	2.0 (6.7)	0.486
Moderate healthy eating practice	4.0 (2.9)	100 (73.5)	21 (15.5)	11 (8.1)	
Healthy eating practice	2.0 (10.5)	13 (68.5)	2.0 (10.5)	2.0 (10.5)	

Data are presented as means ± SD for continuous variables and percentages for categorical variables. Differences between means were tested using One-Way ANOVA, and the Chi-square test was used to examine differences in the prevalence of categorical variables. The bold values indicated that the *P*-value less than 0.05 was considered statistically significant. SD stands for standard deviation. Unhealthy eating practice is defined as a score below 60%, moderate healthy eating practice as a score between 60% and 79%, and healthy eating practice as a score between 80% and 100%.

Table 5 analyzes biochemical markers across different BMI categories (underweight, normal weight, overweight, and obese children). Fasting plasma glucose and hemoglobin levels showed no significant differences across BMI categories. However, IL-6 levels were significantly higher in obese children (*P* = 0.001), indicating a link between obesity and inflammation. Hs-CRP levels were also significantly higher in obese children (*P* = 0.033), with a large proportion of obese children having elevated levels (*P* = 0.001). Adiponectin levels did not differ significantly across BMI categories (*P* = 0.462).

**Table 5:** The relationship between biochemical measurements and body mass index for age categories of the study participants.

Variables	Underweight *n* (%) 6.0 (3.2)	Normal weight *n* (%) 138 (74.6)	Overweight *n* (%) 26 (14.1)	Obese *n* (%) 15 (8.1)	*P* Value
Fasting plasma glucose (mg/dl)					
Mean ± SD	59.6 ± 7.1	65.1 ± 6.9	65.4 ± 6.1	66.0 ± 6.4	0.244
Fasting plasma glucose categories					
Less than 100 mg/dl	6.0 (3.2)	138 (74.6)	26 (14.1)	15 (8.1)	–
Hemoglobin (g/dl)					
Mean ± SD	11.2 ± 0.8	11.8 ± 1.0	12.0 ± 0.9	12.4 ± 0.9	0.056
Hemoglobin categories					
Less than 11.5 g/dl	3.0 (4.1)	56 (76.7)	10 (13.7)	4.0 (5.5)	0.708
11.5 g/dl or more	3.0 (2.7)	82 (73.2)	16 (14.3)	11 (9.8)	
Interlukin_6 (pg/ml)					
Mean ± SD	0.35 ± 0.11	0.43 ± 0.24	0.59 ± 0.23	0.48 ± 0.23	**0**.**001**
Interlukin_6 categories					
Less than 10 pg/ml	6.0 (3.2)	138 (74.6)	26 (14.1)	15 (8.1)	–
Adiponectin (µg/ml)					
Mean ± SD	1.48 ± 1.2	1.02 ± 1.0	0.89 ± 0.9	0.73 ± 0.48	0.462
Adiponectin categories					
Less than 3 µg/ml	5.0 (2.9)	126 (74.1)	24 (14.1)	15 (8.9)	0.576
3 to 30 µg/ml	1.0 (6.7)	12 (80.0)	2.0 (13.3)	0.0 (0.0)	
Hs-CRP (mg/L)					
Mean ± SD	0.65 ± 0.13	1.24 ± 1.5	2.11 ± 1.6	1.38 ± 1.08	**0**.**033**
Hs-CRP categories					
Less than 1 mg/L	6.0 (5.0)	100 (83.3)	6.0 (5.0)	8.0 (6.7)	**0**.**001**
1 to 3 mg/L	0.0 (0.0)	23 (57.5)	12 (30.0)	5.0 (12.5)	
More than 3 mg/L	0.0 (0.0)	15 (60.0)	8.0 (32.0)	2.0 (8.0)	
HDL-c (mg/dl)					
Mean ± SD	38.5 ± 9.4	40.1 ± 8.1	40.4 ± 6.5	38.8 ± 7.5	0.870
HDL-c categories					
≤45 mg/dl	4.0 (3.1)	97 (75.1)	18 (14.0)	10 (7.8)	0.990
More than 45 mg/dl	2.0 (3.6)	41 (73.2)	8.0 (14.3)	5.0 (8.9)	
Triglyceride (mg/dl)					
Mean ± SD	80.3 ± 26.8	84.5 ± 33.8	79.9 ± 24.0	90.3 ± 25.2	0.771
Triglyceride categories					
Less than 75 mg/dl	2.0 (2.3)	70 (80.5)	10 (11.5)	5.0 (5.7)	0.630
75 to 99 mg/dl	3.0 (5.1)	39 (66.1)	11 (18.6)	6.0 (10.2)	
100 mg/dl or more	1.0 (2.6)	29 (74.3)	5.0 (12.8)	4.0 (10.3)	
Fasting insulin (µU/ml)					
Mean ± SD	1.01 ± 0.0	6.65 ± 4.1	17.2 ± 1.4	21.0 ± 1.6	0.149
HOMA-IR					
Mean ± SD	0.16 ± 0.1	1.06 ± 0.6	2.83 ± 0.3	3.47 ± 0.4	**0**.**001**
HOMA-IR categories					
≤3.16	6.0 (3.4)	138 (79.3)	23 (13.3)	7.0 (4.0)	**0**.**001**
More than 3.16	0.0 (0.0)	0.0 (0.0)	3.0 (27.3)	8.0 (72.7)	

Data are presented as means ± SD for continuous variables and as percentages for categorical variables. One-Way ANOVA was used to test differences between means, and the Chi-square test was employed to examine differences in the prevalence of categorical variables. The bold values indicated that the *P*-value less than 0.05 was considered statistically significant. SD stands for standard deviation; HOMA-IR is the Homeostasis Model Assessment of Insulin Resistance; HDL-c is High-Density Lipoprotein Cholesterol; Hs-CRP is High-Sensitivity C-Reactive Protein. HOMA-IR is calculated as (Fasting plasma glucose × Fasting insulin)/405. Insulin resistance is defined as HOMA-IR > 3.16, and no insulin resistance is defined as HOMA-IR ≤ 3.16.

There were no significant differences for HDL-c and triglycerides (*P* = 0.870 and *P* = 0.771), but fasting insulin and HOMA-IR levels were significantly associated with BMI (*P* = 0.001). Overweight and obese children had higher fasting insulin levels, and obese children had the highest HOMA-IR values, indicating insulin resistance. A significant proportion of obese children (72.7%) exhibited insulin resistance, confirming the link between obesity and metabolic changes.

In conclusion, markers of inflammation and insulin resistance, including IL-6, Hs-CRP, fasting insulin, and HOMA-IR, were significantly associated with BMI-for-age categories, with obese children showing higher levels of both inflammation and insulin resistance. Other markers, such as fasting plasma glucose, hemoglobin, adiponectin, HDL-c, and triglycerides, showed no significant associations with BMI.

## Discussion

To the best of our knowledge, this is one of the first study in the Gaza Strip, which investigated the role of inflammation in the relationship between BMI-for-age and insulin resistance among the first-grade students in the Gaza Strip. The main results reveal significant associations between obesity, inflammation, and insulin resistance, which align with existing research linking childhood obesity to inflammatory biomarkers and metabolic issues (36, 37). In addition, this study also highlights socio-economic influences on BMI in the Gaza Strip, particularly the impact of paternal education and urban vs. rural living.

However, beyond understanding the immediate relationship between obesity, inflammation, and insulin resistance, these findings also have significant clinical relevance, particularly concerning the long-term risk of cardiovascular disease. As evidenced by a recent study (38), childhood obesity, characterized by increased BMI, insulin resistance, and hyperinsulinemia, is associated with early cardiovascular morbidity and mortality. Given the high prevalence of obesity, insulin resistance, and inflammation in the pediatric population of the Gaza Strip, it is crucial to implement early prevention strategies to reduce long-term cardiovascular risks. Key approaches include promoting healthy diets and physical activity, especially in urban areas with high access to processed foods, and creating safe spaces for exercise. Targeted interventions should focus on at-risk populations, particularly children from lower socio-economic backgrounds, by educating parents and improving access to healthy food and healthcare. Early screening and monitoring of metabolic risk factors, such as insulin resistance and inflammatory markers, are essential for timely interventions. Additionally, integrating cardiovascular risk education into school programs and establishing community-based initiatives offering resources like healthy food and fitness programs can further support healthier lifestyles across all socio-economic groups.

The study found that two critical inflammatory markers, IL-6 and Hs-CRP, were significantly higher in obese children. These findings are consistent with prior research that has shown childhood obesity is often linked to chronic low-grade inflammation (39, 40). IL-6 and Hs-CRP are well-known indicators of inflammation, and their elevated levels in obese children suggest that obesity is not only a result of poor diet and inactivity but also an inflammatory condition (41). Gokulakrishnan et al. (42) in a previous study have reported similar findings, where higher IL-6 and CRP levels were seen in obese children, with links to insulin resistance and other metabolic problems. The results of the current study support these findings.

The association between increased IL-6 and Hs-CRP levels and obesity in this study supports the hypothesis that inflammation plays a significant role in obesity-related metabolic dysfunctions. This is crucial because adipose tissue inflammation can disrupt insulin signaling, contributing to insulin resistance (43). This study found that 72.7% of obese children had insulin resistance, which aligns with studies like those of Cheng et al. (44), where higher BMI was associated with elevated fasting insulin and HOMA-IR, indicating insulin resistance.

This study also highlighted the role of socio-economic factors in BMI-for-age categories, particularly place of residence and paternal education level. Children from urban areas, especially Gaza City, were more likely to be obese, while those from rural areas like Khan Younis showed higher rates of underweight. These results are in line with previous study that has found urbanization to be a factor in higher obesity rates, often due to greater access to processed foods and more sedentary lifestyles in urban settings (45). Additionally, paternal education level was found to influence children's BMI, with those having less-educated fathers more likely to be obese. This supports the idea that parental education plays a significant role in shaping children's health behaviors and nutrition (46). The low socio-economic status of the families, with most earning below 1974 NIS per month, further suggests that limited access to healthy food, healthcare, and opportunities for physical activity can significantly influence children's BMI. The study found no significant associations between physical activity, eating habits, or breastfeeding practices and BMI categories. While breastfeeding duration has been shown in some studies to help prevent obesity, this study found no significant effect, although there were trends suggesting that exclusive breastfeeding for over six months might reduce overweight prevalence. The lack of a clear correlation between physical activity levels and BMI in this study could be due to confounding factors such as diet, sedentary behaviors, and genetic influences, which may mask the expected benefits of physical activity. Despite increased physical activity, children might still consume unhealthy diets or engage in excessive screen time, undermining the positive effects of exercise. This highlights the complexity of childhood obesity, emphasizing the need for a holistic approach that includes promoting physical activity, improving dietary habits, reducing sedentary behaviors, and addressing socio-economic barriers to healthier living.

Regarding dietary habits, the study found no significant links between specific food groups and BMI categories, although children in the underweight category appeared to have healthier eating practices. This suggests that while diet may not directly influence BMI in this cohort, children who are underweight might be more likely to engage in health-conscious eating behaviors. This observation warrants further exploration, as children in the underweight category may have different eating habits compared to those in the normal or obese categories. In the current study, children with poor appetite showed a slightly higher obesity prevalence. The paradoxical finding that children with poor appetite have a slightly higher obesity prevalence may be due to factors like altered satiety regulation or caregiver misperception. Children with poor appetite may eat irregularly, consuming small amounts of calorie-dense foods, or caregivers may offer larger portions to compensate, leading to excess calorie intake. Hormonal imbalances affecting hunger and fullness signals could also contribute to overeating, despite a lack of initial hunger. This highlights the complex relationship between appetite, eating behavior, and obesity, influenced by both physiological and caregiver factors. Previous research also examines the connection between birth weight and BMI, highlighting how birth weight impacts the risk of childhood obesity, with both low and high birth weight leading to distinct patterns of BMI development as children grow (47, 48).

The study also analyzed biochemical markers, finding no significant differences in fasting plasma glucose and hemoglobin levels across BMI categories. However, insulin resistance was markedly higher in obese children, as indicated by elevated fasting insulin and HOMA-IR levels. These findings are consistent with other research showing that elevated insulin levels are commonly seen in obese children and serve as early indicators of metabolic issues such as type 2 diabetes (49). Conversely, markers like HDL-c, triglycerides, and adiponectin did not differ significantly across BMI categories, suggesting that while obesity is linked to inflammation and insulin resistance, it may not yet cause substantial alterations in lipid metabolism or adipokine levels in children of this age group. While obesity is associated with inflammation and insulin resistance, it may not significantly impact lipid metabolism or adipokine levels in younger children. However, the relationship between inflammation and dyslipidemia is well-established, particularly in obese adults. Chronic mild inflammation can disrupt lipid metabolism, leading to imbalances in lipid profiles and increasing the risk of dyslipidemia and cardiovascular diseases later in life (5). This highlights the potential long-term metabolic consequences of childhood obesity, even before substantial lipid changes occur.

Actually, the cause-and-effect relationship between obesity and inflammation is not yet fully understood, and there appears to be a complex, cyclical interaction between the two. Obesity often leads to a chronic low-grade inflammatory state, with excess adipose tissue producing pro-inflammatory cytokines that impair insulin signaling and contribute to metabolic dysfunction. In turn, this inflammation exacerbates obesity by disrupting metabolic processes, further promoting fat accumulation. This creates a vicious cycle where obesity induces inflammation, and inflammation, in turn, worsens obesity and its associated health risks, making it difficult to break the cycle without targeted interventions (6). Understanding this interplay is crucial for addressing obesity-related health issues.

### Strength and limitations

The main strength of our study lies in being one of the first to explore the role of inflammation in the association between BMI-for-age percentile and insulin resistance among first-grade students in primary governmental schools in the Gaza Strip. However, the study's limitations include the use of non-probability sampling techniques and the inherent limitations of a cross-sectional design, which make it difficult to establish causal associations and limit the generalizability of the findings. Longitudinal studies are needed to track how these metabolic changes progress over time. Additionally, while the study examined socio-economic and lifestyle factors, a more detailed analysis of dietary intake, physical activity levels, and genetic factors could provide deeper insights into the causes of childhood obesity in this population.

## Conclusion

In conclusion, this study supports the notion that childhood obesity is strongly linked to both inflammation and insulin resistance. Elevated levels of IL-6, Hs-CRP, and insulin in obese children underscore the metabolic risks associated with childhood obesity. Socio-economic factors, including place of residence and paternal education, were found to significantly influence BMI-for-age categories, suggesting that efforts to combat childhood obesity should consider these socio-economic factors.

## Data Availability

The raw data supporting the conclusions of this article will be made available by the authors, without undue reservation.
